# Supplementary material for: Whole Genome Characterization and Evolutionary Analysis of G1P[8] Rotavirus A Strains during the Pre- and Post-Vaccine Periods in Mozambique (2012–2017)
Source: Pathogens. 2020 Dec 6;9(12):1026. doi: 10.3390/pathogens9121026 (PMC7762294; doi:10.3390/pathogens9121026)
Supplement: Supplementary file 1 [file pathogens-09-01026-s001.zip › Figure S3. Mozambique Map with the geographical location of study sentinel sites.docx]

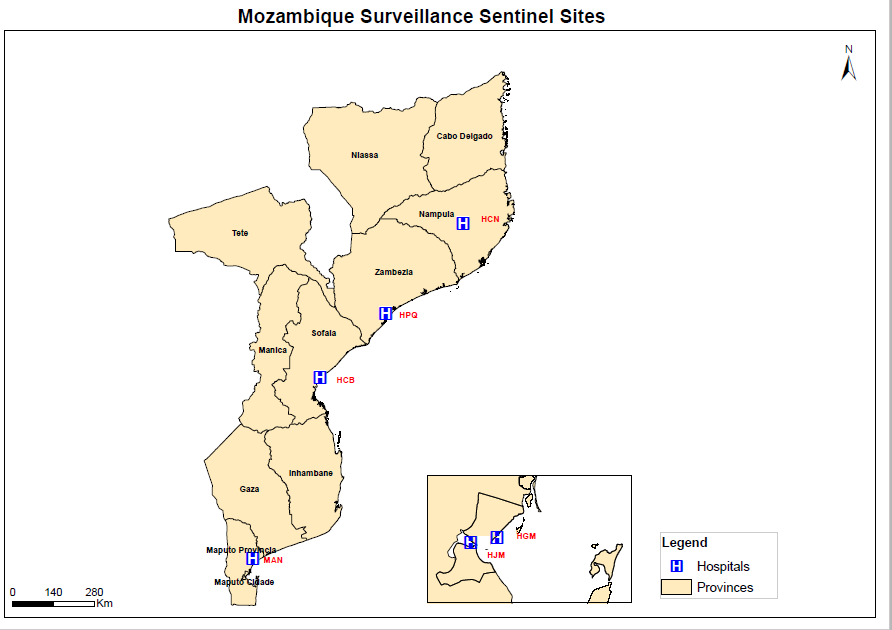


**Figure S3.** Mozambique Map with the geographical location of study sentinel sites. Name of the hospitals are indicated in blue. Hospital Geral de Mavalane, Hospital Geral José Macamo and Hospital Distrital da Manhiça located in Maputo Province; Hospital Central da Beira in Sofala Province, Hospital Provincial da Quelimane in Zambézia Province and Hospital Central de Nampula in Nampula province.

**Table S1**: Genome assembly of Mozambican Wa-like G1P[8] strains from 2012 to 2017. The percentage identity was determined with BLASTn.

**Table S2**: Nucleotide identities of the Mozambican and Rotarix® vaccine strains.

**Table S3**: Mozambican G1P[8] strains.

**Figure S1**: A simplified maximum clade credibility trees (MCC) for the G1 VP7 strains characterized between 1978 and 2017.

**Figure S2**: A simplified maximum clade credibility trees (MCC) for the P[8] VP4 strains characterized between 2000 and 2017.
