# Supplementary material for: Whole Genome Characterization and Evolutionary Analysis of G1P[8] Rotavirus A Strains during the Pre- and Post-Vaccine Periods in Mozambique (2012–2017)
Source: Pathogens. 2020 Dec 6;9(12):1026. doi: 10.3390/pathogens9121026 (PMC7762294; doi:10.3390/pathogens9121026)
Supplement: Supplementary file 1 [file pathogens-09-01026-s001.zip › Table S3 Mozambican G1P[8] strains.docx]

| **Table S3:** Mozambican G1P[8] strains. | | | | | |
| --- | --- | --- | --- | --- | --- |
| **Site Location** | **Hospital** | **Abbreviation** | **Strain Name** | **Rotarix^®^ 1st dose** | **Rotarix^®^ 2nd dose** |
| Maputo | Hospital Distrital da Manhiça | MAN | RVA/Human-wt/MOZ/MAN0033/2012/G1P[8] | NA | NA |
| Maputo | Hospital Geral de Mavalane | HGM | RVA/Human-wt/MOZ/HGM0007/2014/G1P[8] | NA | NA |
| Maputo | Hospital Geral de Mavalane | HGM | RVA/Human-wt/MOZ/HGM0033/2014/G1P[8] | NA | NA |
| Maputo | Hospital Geral de Mavalane | HGM | RVA/Human-wt/MOZ/HGM0048/2014/G1P[8] | NA | NA |
| Maputo | Hospital Geral de Mavalane | HGM | RVA/Human-wt/MOZ/HGM0059/2014/G1P[8] | NA | NA |
| Nampula | Hospital Central de Nampula | HCN | RVA/Human-wt/MOZ/HCN0154/2015/G1P[8] | NA | NA |
| Maputo | Hospital Geral José Macamo | HJM | RVA/Human-wt/MOZ/HJM0338/2015/G1P[8] | NA | NA |
| Maputo | Hospital Geral de Mavalane | HGM | RVA/Human-wt/MOZ/HGM0544/2015/G1P[8] | NA | NA |
| Nampula | Hospital Central de Nampula | HCN | RVA/Human-wt/MOZ/HCN0666/2015/G1P[8] | NA | NA |
| Nampula | Hospital Central de Nampula | HCN | RVA/Human-wt/MOZ/HCN0690/2015/G1P[8] | NA | NA |
| Nampula | Hospital Central de Nampula | HCN | RVA/Human-wt/MOZ/HCN0727/2015/G1P[8] | NA | NA |
| Nampula | Hospital Central de Nampula | HCN | RVA/Human-wt/MOZ/HCN0753/2015/G1P[8] | NA | NA |
| Nampula | Hospital Central de Nampula | HCN | RVA/Human-wt/MOZ/HCN0874/2016/G1P[8] | NV | NV |
| Nampula | Hospital Central de Nampula | HCN | RVA/Human-wt/MOZ/HCN1011/2016/G1P[8] | V | NV |
| Zambezia | Hospital Geral de Quelimane | HPQ | RVA/Human-wt/MOZ/HPQ1152/2016/G1P[8] | NV | NV |
| Nampula | Hospital Central de Nampula | HCN | RVA/Human-wt/MOZ/HCN1181/2016/G1P[8] | NV | NV |
| Nampula | Hospital Geral de Mavalane | HGM | RVA/Human-wt/MOZ/HGM1234/2016/G1P[8] | NV | NV |
| Maputo | Hospital Geral de Mavalane | HGM | RVA/Human-wt/MOZ/HGM1245/2016/G1P[8] | V | V |
| Maputo | Hospital Geral de Mavalane | HGM | RVA/Human-wt/MOZ/HGM1265/2016/G1P[8] | NV | NV |
| Nampula | Hospital Central de Nampula | HCN | RVA/Human-wt/MOZ/HCN1336/2016/G1P[8] | V | V |
| Nampula | Hospital Central de Nampula | HCN | RVA/Human-wt/MOZ/HCN1358/2016/G1P[8] | V | NV |
| Nampula | Hospital Central de Nampula | HCN | RVA/Human-wt/MOZ/HCN1418/2016/G1P[8] | V | NV |
| Nampula | Hospital Central de Nampula | HCN | RVA/Human-wt/MOZ/HCN1531/2017/G1P[8] | NV | NV |
| Nampula | Hospital Central de Nampula | HCN | RVA/Human-wt/MOZ/HCN1552/2017/G1P[8] | V | NV |
| Nampula | Hospital Central de Nampula | HCN | RVA/Human-wt/MOZ/HCN1587/2017/G1P[8] | V | V |
| Nampula | Hospital Central de Nampula | HCN | RVA/Human-wt/MOZ/HCN1602/2017/G1P[8] | V | V |
| Nampula | Hospital Central de Nampula | HCN | RVA/Human-wt/MOZ/HCN1617/2017/G1P[8] | V | V |
| Nampula | Hospital Central de Nampula | HCN | RVA/Human-wt/MOZ/HCN1624/2017/G1P[8] | V | V |
| Nampula | Hospital Central de Nampula | HCN | RVA/human-wt/MOZ/HCN1625/2017/G1P[8] | V | V |
| Maputo | Hospital Geral de Mavalane | HGM | RVA/Human-wt/MOZ/HGM1641/2017/G1P[8] | V | V |
| Maputo | Hospital Geral José Macamo | HJM | RVA/Human-wt/MOZ/HJM1646/2017/G1P[8] | V | V |
| Maputo | Hospital Geral José Macamo | HJM | RVA/Human-wt/MOZ/HJM1650/2017/G1P[8] | V | V |
| Nampula | Hospital Central de Nampula | HCN | RVA/Human-wt/MOZ/HCN1658/2017/G1P[8] | V | V |
| Sofala | Hospital Central da Beira | HCB | RVA/Human-wt/MOZ/HCB1682/2017/G1P[8] | V | V |
| Zambezia | Hospital Geral de Quelimane | HPQ | RVA/Human-wt/MOZ/HPQ1706/2017/G1P[8] | V | V |
| Maputo | Hospital Geral de Mavalane | HGM | RVA/Human-wt/MOZ/HGM1789/2017/G1P[8] | V | V |

NV- not vaccinated

V-vaccinated

NA- not applicable. Refers to the pre-vaccine period.
