# Supplementary figures and images for: Whole Genome Characterization and Evolutionary Analysis of G1P[8] Rotavirus A Strains during the Pre- and Post-Vaccine Periods in Mozambique (2012–2017)
Source: Pathogens. 2020 Dec 6;9(12):1026. doi: 10.3390/pathogens9121026 (PMC7762294; doi:10.3390/pathogens9121026)

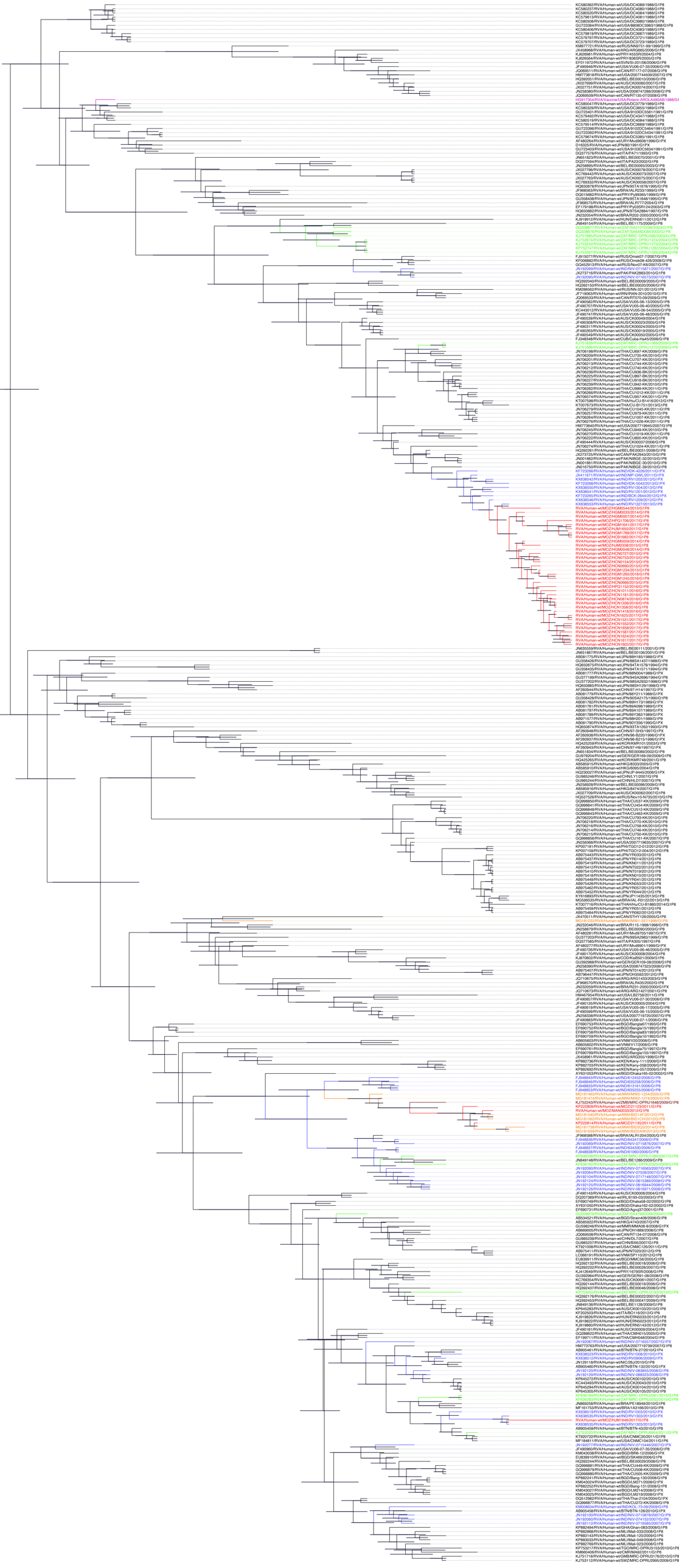

Supplement: Supplementary file 1 [file pathogens-09-01026-s001.zip › Figure S1_378_G1P8_Global_VP7_HKYG_200M_run2_MCC_tree.pdf]

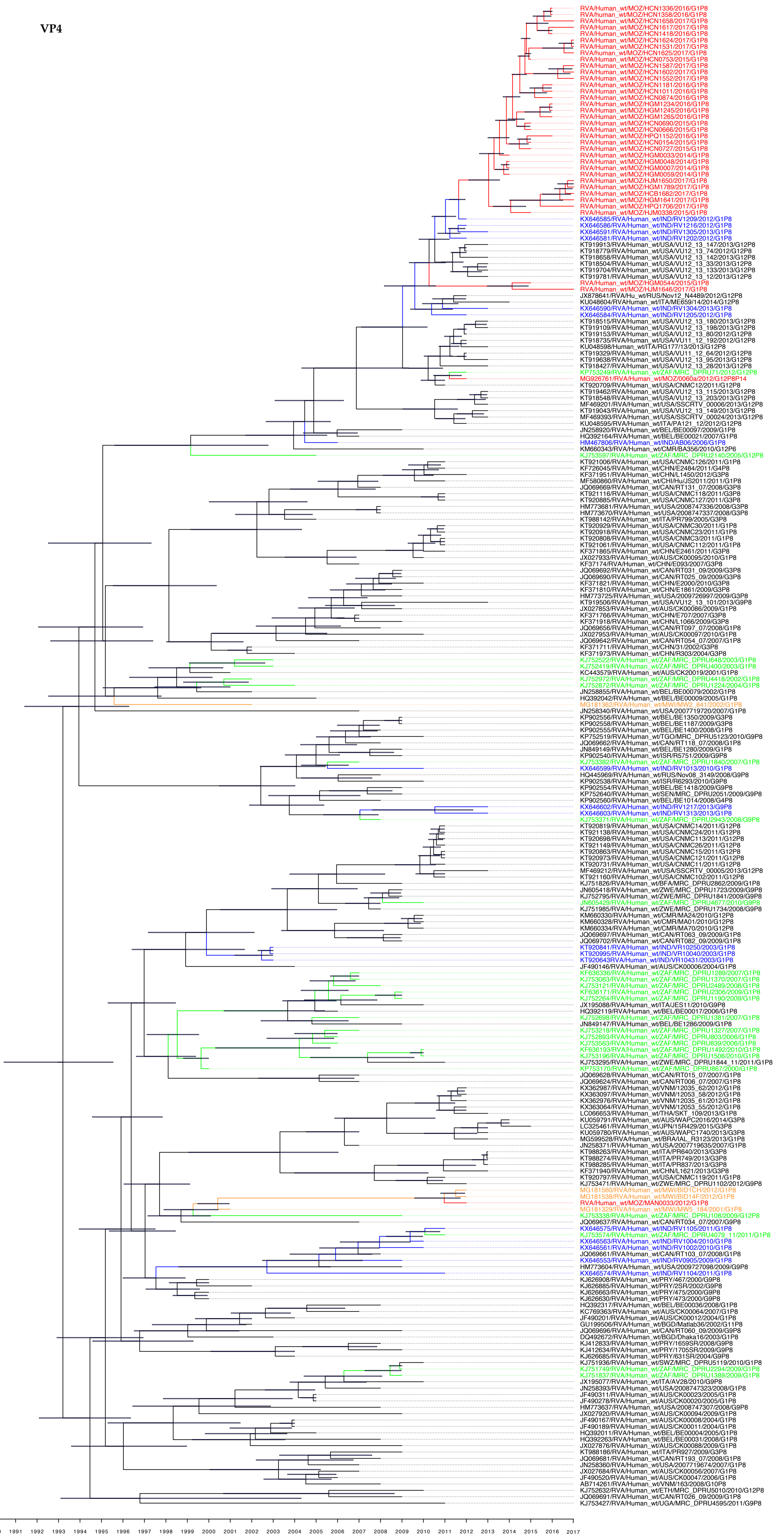

Supplement: Supplementary file 1 [file pathogens-09-01026-s001.zip › Figure S2_235_Global_VP4_GTRG_GMRF_200M_run1_MCC_tree.pdf]
